# Chronic kidney disease, worsening renal function and outcomes in a heart failure community setting: A UK national study^☆^

**DOI:** 10.1016/j.ijcard.2018.04.090

**Published:** 2018-09-15

**Authors:** Claire A. Lawson, J.M. Testani, M. Mamas, K. Damman, P.W. Jones, L. Teece, U.T. Kadam

**Affiliations:** a Leicester Diabetes Centre, Leicester University, UK; b Keele Cardiovascular Research Group, Centre for Prognosis Research, Institute of Primary Care and Health Sciences, University of Keele, Stoke-on-Trent, UK; c Yale University, New Haven, CT, United States; d University of Groningen, University Medical Center, Groningen, The Netherlands; e Faculty of Medicine and Health Sciences, Keele University, England, UK

**Keywords:** aOR, adjusted Odds Ratio, BMI, Body mass index, BP, Blood pressure, CI, Confidence Interval, CKD, Chronic kidney disease, eGFR, Estimated glomerular filtration rate, CPRD, Clinical Practice Research Datalink, HF, Heart Failure, IHD, Ischaemic heart disease, Heart failure, Chronic kidney disease, Worsening renal function, Comorbidity, Hospitalisation, Death, Population based

## Abstract

**Background:**

Routine heart failure (HF) monitoring and management is in the community but the natural course of worsening renal function (WRF) and its influence on HF prognosis is unknown. We investigated the influence of routinely monitored renal decline and related comorbidities on imminent hospitalisation and death in the HF community population.

**Methods:**

A nested case-control study within an incident HF cohort (*N* = 50,114) with 12-years follow-up. WRF over 6-months before first hospitalisation and 12-months before death was defined by >20% reduction in estimated glomerular filtration rate (eGFR). Additive interactions between chronic kidney disease (CKD) and comorbidities were investigated.

**Results:**

Prevalence of CKD (eGFR<60 ml/min/1.73m^2^) in the HF community was 63%, which was associated with an 11% increase in hospitalisation and 17% in mortality. Both risk associations were significantly worse in the presence of diabetes. Compared to HF patients with eGFR,60–89, there was no or minimal increase in risk for mild to moderate CKD (eGFR,30–59) for both outcomes. Adjusted risk estimates for hospitalisation were increased only for severe CKD(eGFR,15–29); Odds Ratio 1.49 (95%CI;1.36,1.62) and renal failure(eGFR,<15); 3.38(2.67,4.29). The relationship between eGFR and mortality was U-shaped; eGFR, ≥90; 1.32(1.17,1.48), eGFR,15–29; 1.68(1.58,1.79) and eGFR,<15; 3.04(2.71,3.41). WRF is common and associated with imminent hospitalisation (1.50;1.37,1.64) and mortality (1.92;1.79,2.06).

**Conclusions:**

In HF, the risk associated with CKD differs between the community and the acute HF setting. In the community setting, moderate CKD confers no risk but severe CKD, WRF or CKD with other comorbidities identifies patients at high risk of imminent hospitalisation and death.

## Highlights

• The study was based on 50,114 heart failure patients from UK general practices.
• Risk associated with CKD in the community is limited to the severest dysfunction.
• CKD interacts with diabetes, significantly worsening outcome risk.
• WRF is associated with high risk of imminent hospitalisation or mortality.
• Routine renal function monitoring in the community offers a simple prognostic tool.

## 1 Introduction

Chronic kidney disease (CKD) is a prevalent comorbidity in heart failure (HF) affecting approximately half of patients in the general population [1]. In acute HF, reduced renal function consistently doubles the risk of mortality or more depending on the severity of impairment [2]. Worsening renal function (WRF) is a common feature in the varying HF course and treatment, which further increases mortality risk [3]. Much of the current WRF evidence relates to short term change during acute HF decompensation in selected samples [3]. However, the pathophysiology, presentation and treatment of WRF differs substantially for acute and chronic HF [4]. In acute HF, WRF usually occurs as a consequence of intensive, sudden or rapid changes in fluid balance. In contrast, WRF in chronic HF is more likely to be over weeks or months [5] which may have considerably different prognostic implications, but here the evidence is limited.

In the community setting, HF studies have focused mainly on WRF during optimisation of HF modifying drugs [[6], [7], [8]]. Short term changes during HF drug intensification however is an unreliable indicator of WRF, instead reflecting an appropriate response to treatment [6,9]. Treatment-related WRF can be temporary and clinically patients may improve in the longer term [10]. Current HF population studies investigating chronic WRF are limited to reduced ejection fraction in small [11] or trial samples [12,13]. The community setting in contrast represents the range of patients with reduced and preserved ejection fraction or with the comorbidities commonly encountered in the HF general population. Notably CKD is associated with hypertension, ischemic heart disease and diabetes, through both patho-physiology and drug treatment, and these combined together might increase the prognostic risk even more in this population.

In UK primary care, renal function is routinely monitored in HF patients. Through linkage to hospitalisations and mortality data, our objectives were (i) to investigate the association between renal dysfunction, its longer term change and imminent hospital admission and death in the heart failure community setting and (ii) the influence of other comorbidities on these relationships.

## 2 Methods

The Clinical Practice Research Datalink (CPRD) is a large validated database of anonymised primary care medical records covering approximately 11% of the UK population [14]. Data includes patient demographic information, clinical diagnoses, prescriptions, laboratory tests and lifestyle information. Linkage to admissions data based on all Hospital Episode Statistics (HES) and mortality data is available for consenting practices (~60%). Use of the CPRD database was under licence (protocol 12_162) with approval granted from the Independent Scientific Advisory Committee.

### 2.1 Study population

Incident HF patients aged over 40 years who had minimum 3-years of quality clinical data recording prior to study entry, were selected by a first HF diagnostic code applied in their CPRD clinical record between January 1st 2002 and March 1st 2012 (Supplementary Table 1 for code set). The HF code set was validated by clinicians and shown to have high validity in CPRD [15]. Time in follow-up was from the first HF code to either the date of the patient transferring out of practice, the index event, death or the study end (1st January 2014).

### 2.2 Selection of cases and controls

We conducted two separate nested case-control studies within the incident HF cohort for the outcomes of: (i) all-cause mortality and (ii) all-cause first hospitalisation after HF diagnosis. A nested case-control design with density sampling of controls was applied to account for the varying nature of renal function, pharmacology (e.g. neurohormonal antagonists) and other clinical factors that vary over time. Using this approach, renal function and all covariates are measured for each case and matched controls, at the same time during follow-up and prior to every event. Controls are eligible to be selected multiple times and later as a case, meaning that changes to exposure status are captured along the entire follow-up period. This closely resembles the programming statements approach to Cox-regression with time varying covariates and produces unbiased estimates of hazard ratios [16,17].

For each case, up to four controls were randomly sampled from the HF cohort who were still at risk of the event. Matching was by HF index date (±1 month) and duration of follow-up and the controls were assigned the index date of the case.

### 2.3 Measurement of renal function

Estimated glomerular filtration rate (eGFR) was calculated using the Chronic Kidney Disease Epidemiology Collaboration (CKD-EPI) formula [18]. Renal function was based on the most recent eGFR measure before the outcome dates excluding values recorded >6-months before hospitalisation (median interval 51 [20 to 100] days) and 12-months before mortality (median interval 84 [32 to 171] days), as the study focus was on short-term outcomes. CKD was defined as eGFR <60 ml/min per 1.73 m2. Patients were categorised into six groups recommended by Kidney Disease: Improving Global Outcomes (KDIGO) [19]. The categories of renal function were: ≥90 (“high”, stage 1), 60–89 (“mild”, stage 2), 45–59 (‘mild to moderate’, stage 3a), 30–44 (‘moderate to severe’, stage 3b), 15–29 (“severe”, stage 4) and < 15 (“kidney failure” or dialysis, stage 5).

### 2.4 Worsening Renal Function (WRF)

WRF was calculated as the difference between two eGFR values separated by at least 3-months as a proportion of the baseline value. Previous values exceeding 12-months before the most recent value for hospitalisation (median interval 118 [62 to 208] days) and 2-years for mortality were excluded (median interval 301 [227 to 393] days). WRF was then adjusted for the interval time to calculate change in eGFR over 6-months for hospitalisation and 12-months for mortality. The time-window for mortality was chosen to investigate longer term eGFR change and to maximise the potential of available data, however change over a shorter time was likely to be more clinically relevant as a potential prognostic indicator for hospitalisation. WRF was defined as a > 20% reduction in eGFR [20] and moderate reduction as 6–20% decrease. We also included a category of any increase in eGFR. All categories were compared to a reduction of 5% or less as the reference group.

### 2.5 Measurement of confounding

Confounders measured most recent to outcome dates were age, sex, Index Multiple Deprivation (IMD) [21], body mass index (BMI), smoking, alcohol, cholesterol, haemoglobin, blood pressure (BP), hypertension, ischemic heart disease (IHD), previous myocardial infarction, atrial fibrillation, diabetes and chronic obstructive pulmonary disease [1]. The IMD score was ranked into quintiles from lowest to highest deprivation; smoking and alcohol were categorised into ‘current, previous or never’ and all other measures retained as continuous variables. Drug measures for aspirin, renin angiotensin aldosterone system (RAAS) drugs (angiotensin converting enzyme inhibitors, angiotensin II receptor blockers), beta-blockers and diuretics were also extracted in the 4-month time-window before the matched dates. For the hospital linked cohort, models were also adjusted for a prior hospital admission in the 0–3 months, 3 to 6 months or 6 to 12-months before the HF index date.

### 2.6 Statistical analysis

Tables on case or control status for both outcomes and presence of WRF are presented to compare study measures. To build the multivariable models, linearity was investigated using likelihood ratio tests and quadratic extensions were added for any non-linear continuous variables. For correlated variables with coefficient > 0.5, the most clinically relevant one was selected. Multiple imputations using matching variables and full-conditional specification were performed for the missing data using MI Impute procedure in Stata version 13, and analyses were combined using Rubin's rules [22].

Conditional logistic regression was used to estimate odds ratios (OR) for the two outcomes comparing HF patients with CKD compared to those without, adjusting for all confounders. To examine the interaction between CKD and hypertension, diabetes or ischemic heart disease in HF, we tested whether their observed combined effect (CKD + comorbidity) on an outcome was more or less than the sum of their separate effects. Using the Rothman approach [23], for each CKD and comorbidity pair, we created dummy variables for the four possible joint exposure combinations (absence/absence [reference], presence/absence, absence/presence and presence/presence) (Supplementary Table 2). We then performed conditional logistic regression for the dummy variables for each exposure pair in turns. We calculated two measures of additive interaction with 95% confidence intervals; the relative excess risk due to the interaction (RERI) and the Synergy index (S). The RERI is the difference between the expected risk associated with joint exposure (calculated by adding the individual observed risks) and the observed risk of joint exposure. The Synergy index (S) is the proportion of excess risk from joint exposure in the presence of interaction relative to if there was no interaction. Interaction is indicated where RERI ≠ 0 and/or synergy index ≠ 1 [24].

Next, we compared different CKD stages to reference stage 2 (60–89 ml/min/1.73 m2). We chose this reference group as opposed to eGFR ≥90 due to the normal renal decline in older age. WRF and other categories of renal function change were then compared to the eGFR reference group with <5% decrease. In sensitivity analyses, the models investigating renal change were further adjusted for the first and second eGFR measure used in the change calculation and the mortality models were further adjusted for deprivation.

## 3 Results

### 3.1 HF population characteristics

There were 50,114 incident HF patients, of whom 26,729(53%) died during a median follow-up of 2.6[IQR 0.8–5.0] years. Of the sample with linked hospital data (*n* = 30,061), 24,339(81%) experienced a first hospitalisation after their HF index date during a median follow-up of 82[IQR 12–435] days. Cases for both outcomes were older, with more comorbidities and less HF modifying drugs during follow-up, with a lower BMI, cholesterol, haemoglobin and blood pressure (Table 1). Among the hospitalisation cases, 66% had CKD compared to 59% of controls and 20% had a prior hospital admission in the 3 months before their heart failure index date, compared to 4% of controls. In the mortality cohort, CKD was present in 75% of cases and 62% of controls.

**Table 1:** HF population characteristics of cases and controls by outcomes.

Characteristics	Hospitalisation			Mortality		
			
	Cases (*n* = 24,339)	Controls (*n* = 86,450)	Missing n(%)	Cases (*n* = 26,729)	Controls (*n* = 106,916)	Missing n(%)
Age, years	79[72–85]	78[70–84]	–	83[70–84]	78[76–88]	–
Women	11,388(46.8)	42,416(49.1)	–	12,974(48.5)	48,758(45.6)	–
IMD quintile 1 (least)	4659(19.1)	17,834(20.6)		3064(18.7)	12,844(20.4)	
2	5597(23.0)	20,928(24.2)		3708(22.6)	14,381(22.8)	
3	5142(21.1)	17,794(20.6)		3570(21.8)	13,096(20.8)	
4	5046(20.7)	17,459(20.2)		3427(20.9)	13,024(20.7)	
5 (most)	3825(15.7)	12,163(14.1)		2613(16.0)	9656(15.3)	
BMI(Kg/m2)	26.8[23.5–30.6]	27.3[24.2–31.4]	9.6	25.4[22.1–29.3]	27.3[24–31.3]	8.9
Cholesterol(mmol/L)	4.6 ± 1.2	4.7 ± 1.2	17.7	4.4 ± 1.2	4.5 ± 1.2	14.4
Hb(g/dL)	12.9 ± 1.9	13.4 ± 1.6	11	12.2 ± 2.0	13.1 ± 1.8	11.6
Systolic BP(mmHg)	134.1 ± 21.6	136.0 ± 19.8	0.5	126.9 ± 22.3	132.5 ± 19.9	0.6
Diastolic BP(mmHg)	74.7 ± 12	75.9 ± 11.0	0.5	71.1 ± 12	73.9 ± 11.1	0.6
Beta blocker	8893(36.5)	35,574(41.2)	–	12,171(45.5)	62,050(58)	–
ACEi	12,477(51.3)	51,430(59.5)	–	12,207(45.7)	62,166(58.1)	–
ARB	3722(15.3)	15,602(18.1)	–	3170(11.9)	19,583(18.3)	–
Spironolactone or Eplerenone	3203(13.3)	10,042(11.6)		5610(21.0)	18,253 (17.1)	–
Diuretics	17,023(69.9)	62,837(72.7)	–	21,574(80.7)	81,709(76.4)	–
Hypertension	13,895(57.1)	48,607(56.2)	–	15,403(57.6)	62,055(58.0)	
IHD	11,197(46)	33,570(38.8)	–	13,394(50.1)	51,761(48.4)	–
Atrial fibrillation	8470(34.8)	27,145(31.4)		10,210(38.2)	39,238(36.7)	–
Previous MI	6171(25.4)	17,499(20.2)	–	7509(28.1)	28,489(26.7)	–
COPD	3230(13.3)	8673(10.3)	–	4630(17.3)	13,848(13.0)	–
DM	5577(22.9)	15,714(18.2)	–	6714(25.1)	25,248(23.6)	–
Smoking status			2.4			1.9
Yes	2965(12.1)	8831(10.2)		3066(11.8)	11,671(11.1)	
No	10,899(44.8)	40,875(47.3)		12,177(46.7)	48,350(46.0)	
Ex	9854(40.5)	34,731(40.2)		10,829(41.5)	45,072(42.9)	
Alcohol status			10.1			9.2
Yes	15,678(64.4)	57,882(67.0)		15,744(66.3)	68,370(70.0)	
No	5161(21.2)	17,006(19.7)		6730(28.3)	24,600(25.2)	
Ex	955(3.9)	2975(3.4)		1270(5.4)	4652(4.8)	
Hospital admission during 1-year prior to CPRD HF index date				N/A	N/A	
0–3 months before	5085(20.9)	3578(4.1)				
3–6 months before	2509(10.3)	5878(6.8)				
6 to 12 months before	2918(12.0)	10,012(11.6)				
eGFR(ml/min/1.73 m2)	52.5 ± 19.9	56.4 ± 18.3	34.7	46.3 ± 20.7	54.9 ± 19.6	20.7
CKD (eGFR<60)	10,429(65.6)	33,523(59.3)	–	15,821(74.9)	52,388(61.7)	–
CKD stage^a^						
1: ≥90	547(3.4)	2312(4.1)		464(2.2)	3627(4.3)	–
2: 60–89	4816(30.9)	20,672(36.6)		4826(22.9)	28,896(34.0)	–
3A: 45–59	4456(28.0)	17,403(30.8)		4927(23.3)	24,294(28.6)	–
3B: 30–44	3836(24.1)	12,328(21.8)		5909(28.0)	19,774(23.3)	–
4: 15–29	1796(11.3)	3571(6.3)		3976(18.8)	7333(8.6)	–
5: <15	341(2.2)	221(0.4)		1009(4.8)	987(1.2)	–
eGFR change			50.6			31.9
0–5% decrease (Reference group)	1429(11.6)	6019(14.2)	–	1912(10.5)	11,522(15.9)	–
>20% decrease (WRF)	3166(25.7)	8083(19.0)	–	5613(30.7)	13,096(18.0)	–
6–20% decrease	2757(22.4)	10,211(24.1)	–	3707(20.3)	17,637(24.3)	
Any % increase	4965(40.3)	18,142(42.7)	–	7065(38.6)	30,419(41.9)	–

Data are number patients(%) or mean ± standard deviation or median[IQR]. IMD, index multiple deprivation(1 = least deprived, 5 = most deprived); BMI, body mass index; Hb, haemoglobin; BP, blood pressure; ACEi, angiotensin-converting enzyme inhibitor; ARB, angiotensin receptor blocker; IHD, ischaemic heart disease; MI, myocardial infarction; COPD, chronic obstructive pulmonary disease; eGFR, estimated glomerular filtration rate.

a National Kidney Foundation Kidney Disease Outcomes Quality Initiative(KDOQI) guidelines. For all-cause mortality, change was calculated over a year before the match date using the most recent value (up to a maximum of 1 year) and a second value between 3 months and 2 years earlier. For all-cause hospital admission, change was calculated over 6-months before the match date using the most recent value (up to a maximum of 6-months) and a second value between 3-months and 1 year earlier.

### 3.2 Chronic kidney disease and outcomes

**Fig. 1:**
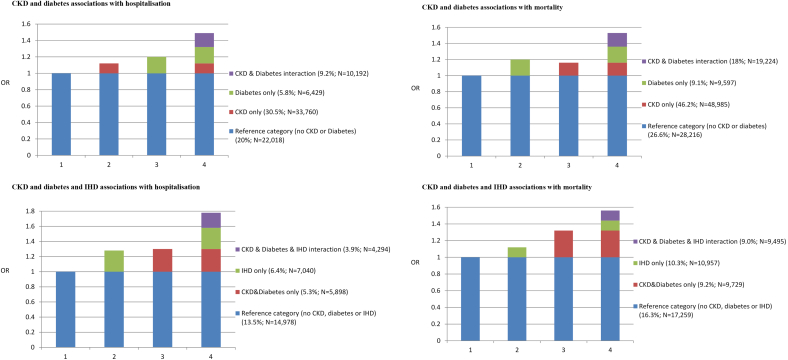
Additive interaction between CKD and comorbidity combinations -The blue block (bars 1 to 4) represents the risk in the HF reference group without any of the specified comorbidities present. -The green and the red blocks (bars 2 to 4) show the risk associated with the specified comorbidities in addition to the reference group. -Bar 4 displays the risk when all of the specified comorbidities are present. The stacked red and green blocks represent the risk that is expected when the specified comorbidities are present (or added) together. The purple block shows the actual or observed risk when the specified comorbidities are present together and represents the risk due to interaction of the comorbidities. This means that the observed combined effect of the comorbidities on the outcome was more than the sum of their separate effects.

The relative risk of hospitalisation was 11% higher and mortality 17% higher for HF patients with CKD compared to those without (Table 2). The combined effect of CKD and DM was significantly higher than would be expected by their simple co-occurrence (Supplementary Table 3). Compared to HF patients without CKD or diabetes, the increase in hospitalisation risk associated with CKD without diabetes was 12% and with diabetes without CKD was 20%. The expected increase in risk in the presence of both comorbidities was therefore (12 + 20 = 32%). The actual observed increase in risk for HF patients with both CKD and diabetes compared to the reference group was 49%. This gave a significant relative excess risk due to interaction (RERI) between CKD and diabetes of 17% (RERI 0.17; 95%CI 0.04,0.29) (see Fig. 1). The synergy index (S) determines the relative proportion of risk due to interaction relative to if there was no interaction present. The S of 1.52 (95% CI 1.05,2.2) can be interpreted as 52% excess risk from CKD and diabetes interaction relative to if there was no interaction present. The higher risk associated with CKD and diabetes than would be expected was consistent for mortality and further compounded by the presence of ischemic heart disease. The observed risk of hospitalisation and mortality associated with all three comorbidities was 20% and 13% higher than would be expected by their co-occurrence, respectively.

**Table 2:** Associations between CKD severity, worsening renal function and outcomes in community HF patients.

	First hospitalisation		All-cause mortality	
	Odds ratio (95% CI)		Odds ratio (95% CI)	
	Unadjusted	Adjusted	Unadjusted	Adjusted
No CKD (eGFR ≥60)(ref)	1.0	1.0	1.0	1.0
CKD (eGFR <60)	1.31(1.26–1.36)	1.11(1.05–1.16)	1.87(1.81–1.94)	1.17(1.12–1.22)
CKD stages^a^				
eGFR mL/min/1.73m^2^				
Stage 1 (60–89)(ref)	1.0	1.0	1.0	1.0
Stage 2 (≥90)	0.95(0.86–1.06)	0.95(0.85–1.08)	0.76(0.69–0.85)	1.32(1.17–1.48)
Stage 3a (45–59)	1.08(1.02–1.13)	1.02(0.97–1.08)	1.23(1.17–1.28)	0.99(0.94–1.04)
Stage 3b (30–44)	1.29(1.22–1.36)	1.10(1.03–1.17)	1.80(1.72–1.88)	1.16(1.10–1.22)
Stage 4 (15–29)	2.09(1.95–2.25)	1.49(1.36–1.62)	3.23(3.07–3.40)	1.68(1.58–1.79)
Stage 5 (<15)	6.24(5.09–7.65)	3.38(2.67–4.29)	6.25(5.65–6.91)	3.04(2.71–3.41)

*Worsening renal function*^b^				
0–5% decrease(ref)	1.0	1.0	1.0	1.0
>20% decrease (WRF)	1.71(1.58–1.86)	1.50(1.37–1.64)	2.64(2.48–2.80)	1.92(1.79–2.06)
6–20% decrease	1.16(1.07–1.26)	1.11(1.02–1.21)	1.27(1.19–1.35)	1.12(1.04–1.20)
Any % increase	1.20(1.11–1.29)	1.13(1.04–1.22)	1.40(1.32–1.48)	1.22(1.15–1.31)

Adjusted for current age, gender, Hb and Hb^2^, BMI and BMI2, beta blocker, ACEi, ARB, spironolactone or eplerenone, diuretic, IHD, previous MI, hypertension, AF, COPD, cholesterol, systolic BP and systolic BP^2^, alcohol use and smoking. Hospitalisation further adjusted for prior hospitalisation over 3,6 or 12-months and deprivation.

a National Kidney Foundation Kidney Disease Outcomes Quality Initiative(KDOQI) guidelines.

b For hospitalisation, renal change was calculated over 6-months before the match date using the most recent value (up to a maximum of 6-months) and a second value between 3-months and 1 year earlier. For mortality, renal change was calculated over a year before the match date using the most recent value (up to a maximum of 1 year) and a second value between 3 months and 2 years earlier.

### 3.3 CKD stages and outcomes

**Fig. 2:**
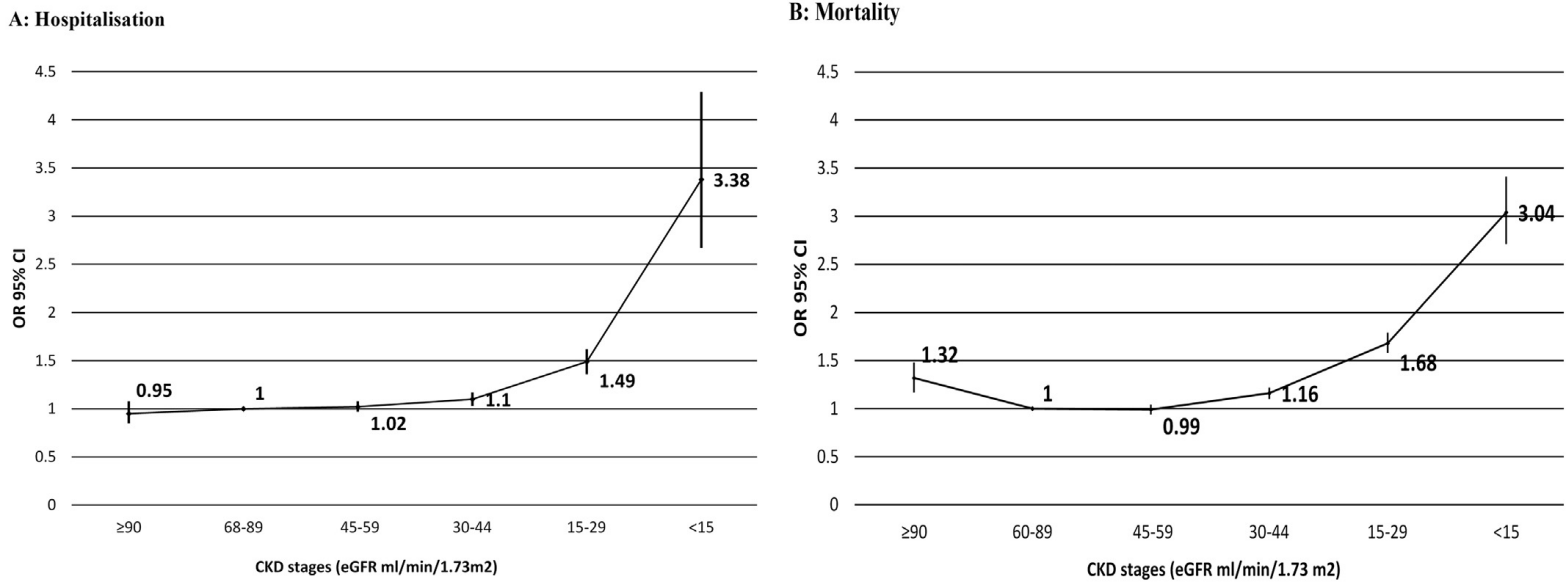
Adjusted associations between eGFR categories and outcomes.

Compared to the eGFR reference group, there was a graded increase in risk for both outcomes with increasing CKD stage, starting at stage 3b and steeply increasing for CKD stage 4 and 5 (Table 2). Of the hospitalisation cases, 14% had CKD Stage 4/5 compared to 7% controls which corresponded to an adjusted relative risk increase of hospitalisation of 49% for stage 4 and 238% for stage 5. Presence of CKD stage 4/5 in 24% of mortality cases compared to 10% of controls, corresponded to increases in relative risk of death of 68% for CKD stage 4 and 204% for stage 5. eGFR values between 45 and 59 ml/min/1.73 m2 were not associated with increased risk of either outcome. This group were prescribed higher levels of RAAS drugs, similar to the eGFR reference group (supplementary tables 4 and 5). Whilst there was a J-shaped relationship between CKD stages and first hospitalisation, the relationship with mortality was U-shaped (Fig. 2) with a 32% increase in relative risk for stage 1 CKD (eGFR ≥90) compared to stage 2 (eGFR 60–89 ml/min/1.73 m2). In the mortality cohort, compared to the reference group with normal renal function, the CKD stage 1 group were much younger (median age 61 v. 75 years), mostly male (74% v. 62%) with a high BMI (median 29 v. 27) and higher levels of smoking (27% v. 13%) and diabetes (32% v. 25%) (Supplementary tables 4). When the CKD stage 1 group were further stratified by mortality outcome (supplementary table 6), the case group were younger (median age 65 v. 75 years) and had a much higher prevalence of COPD (33% v. 16%), diabetes (32% v. 25%) and smoking (35% v. 13%), a lower BMI (26 v. 27) and much lower levels of prescribed RAAS drugs (59% v. 76%) compared to the normal renal function reference group.

### 3.4 WRF and outcomes

WRF occurred in 26% of HF patients over 6-months before hospitalisation compared to 19% of controls and the respective figures were 31% and 18% in the year before death. Patients with WRF were more likely to be female, have hypertension or diabetes, and prescribed more diuretics than those without WRF (Supplementary Table 7). In adjusted stepwise analyses, high starting and low ending eGFR and younger age were associated with WRF before both outcomes. In addition, there were independent associations between WRF and male status, prescribed ACEi or ARB or diuretic, ischemic heart disease, and lower BP values for admission and lower cholesterol and diabetes for mortality (*p* < 0.01) (not shown).

Compared to those with stable renal function, HF patients with WRF over 6-months had a 50% relative increase in risk of imminent hospitalisation (Table 2**)**. This was higher for mortality with WRF over a year associated with a 92% higher relative risk of death compared to those with stable renal function. More moderate decrease in renal function (6–20%) was only associated with a small increase in risk of either outcome. Theses associations were independent of the starting or subsequent eGFR values (Supplementary Table 8). Increase in renal function was also associated with a 13–22% increase in relative risk of hospitalisation and death respectively. This group were prescribed less RAAS inhibitors (74% v. 78%) and had more hypertension (62% v. 60%), AF (39% v. 37%) and diabetes (29% v. 27%) and less IHD (45% v. 56%) than the stable renal function group (supplementary table 7).

## 4 Discussion

In a national HF population-based cohort of over 50,000 community patients, CKD was common, but did not increase the risk of hospitalisation or mortality unless it was severe, or kidney failure was present. However, the risk associated with CKD increased significantly in the presence of diabetes and ischemic heart disease. Worsening renal function was also common, occurring in a quarter of all HF patients and significantly increased the risk of imminent hospitalisation within 6-months or death within 12 months. These findings provide the key prognostic evidence for identifying high risk HF comorbid groups in the community where most patients are routinely managed and receive ongoing care.

There are four key clinical implications of our findings. First, whilst the high prevalence of CKD in the community HF population was comparable to hospital [25] and other specialist care settings [26], it did not convey the same risk of poor outcomes found in more acute settings where HF decompensation is common [1,27]. In the community setting, mild to moderate renal dysfunction (CKD stage 3a) did not confer risk of hospitalisation or death and risks only began to rise steeply at eGFR levels below 30 ml/min/1.73 m2. Of note, over 75% of HF patients with stage 3a and 3b CKD were prescribed ACEi or an ARB, similar to those without CKD and similar to specialist HF clinics [28]. These drugs are recommended in heart failure with reduced ejection fraction (HFrEF) even in the presence of moderate renal dysfunction [20]. Whilst we did not have ejection fraction data available, the high proportion of prescribing indicates good adherence to HF guidelines and that patients with mild to moderate renal deterioration are well managed in the community.

Second, the associations between CKD stages and mortality had a more complex U-shape relationship, with an increased risk in CKD stage 1 compared to CKD stage 2. Whilst other community samples have found similar increases in mortality risk at eGFR ≥90 [29,30], other low ejection fraction studies focusing on cardiovascular specific outcomes have reported a linear relationship between decreasing renal function and mortality risk [13,31]. This high eGFR and mortality risk paradox reported in general populations is thought to represent a false estimation of kidney function due to loss of muscle mass linked to frailty and predominating more in comorbid groups [32,33]. In our study the high eGFR mortality case group, compared to the HF group with normal renal function, were predominantly younger male smokers with a lower BMI and much more likely to have COPD or diabetes, with lower prescribing of RAAS drugs. This profile points to the possibility of hyperfiltration in early diabetic nephropathy [34], or frailty in end stage respiratory or cardiac disease. Our community findings show that very low and high eGFR levels might be prognostic indicators of a more severe HF group, but that high eGFR levels need to be interpreted cautiously in the context of comorbidity and associated frailty.

Third, our study provides new evidence on the interaction between CKD and other cardio-renal comorbidities. The risk of both outcomes associated with CKD and diabetes was significantly greater than would be expected and this was further compounded by ischemic heart disease (IHD). CKD is a well-known risk factor for IHD which can lead to further renal deterioration [35] and diabetic nephropathy is one of the most frequent causes of end-stage renal disease [36]. These findings are important because not only do these comorbidities commonly co-occur but they exacerbate each other, providing the potential for intensified progression. CKD and diabetes was present in 18% of patients and over half of these also had concomitant IHD which indicates important and high risk groups in HF. Our findings indicate an important need for shared management and optimisation of therapies for the most common comorbid disease combinations.

Fourth, WRF occurred in 21% of the community HF patients and was significantly associated with increased risk of imminent 6-month hospitalisation or 12-month mortality. Part explanation of these associations might relate to the underlying hemodynamic status of the HF with worsening renal function acting as a pseudo marker of worsening cardiac function. However, the dose-response relationship between increasing severity of WRF and higher risk and the adjustment for key factors including relevant drugs and baseline renal function, suggests independent associations between WRF and outcomes. Improving renal function in 40% of the HF sample was also associated with poorer outcomes, which is consistent with comparable evidence in acute decompensated HF [37]. Improved function might reflect treatment with diuretics following right sided cardiac dysfunction and venous congestion but this finding requires further investigation in the community setting.

### 4.1 Strengths and limitations

Our study is one of the largest to date to investigate longitudinal renal function and change in a population-based community cohort of newly diagnosed HF patients. The easily accessible routine monitoring measures of CKD and change offers an important tool for risk stratification in the community and potential trigger of earlier interventions. Key strengths include the large national HF sample, linkage to hospital data and the use of density sampling that provided the method to measure WRF in specific time-windows before hospitalisation and death.

Routinely collected data can be subject to measurement bias [38], but the CPRD is used as a clinically validated population-based epidemiological database globally [14]. The study focus was on HF prognosis in the non-specialist community setting, so the definition of HF was based on clinical coding, the quality for which is high [15]. However, lack of ejection fraction or brain natriuretic peptide data in the CPRD means that further validation of the study findings in different HF phenotype groups would be required. Lack of HF severity indicators in the CPRD such as ejection fraction and NYHA class means that it was not possible to disentangle the effects of renal from cardiac dysfunction. In considering confounders, comorbidities were based on clinical recording which can be subject to misclassification leading to under-ascertainment. That said, any such misclassification is likely to bias the associations towards the null value. There was also some missing data for measures such as BMI and smoking. We used multiple imputations for some of these missing data, but we did not impute eGFR values as this was our exposure of interest. Whilst 80% of patients had at least one eGFR measure prior to the case-control match dates, only approximately half of the patients had two measures available and this means that some exposure information was excluded from our analyses. HF management will have also changed in the 12-year time window of the database, and to minimise the potential effect of such changes, in our study design case and controls were matched on calendar time and eGFR and confounders were measured before the matched dates.

## 5 Conclusions

Our large scale study has shown that in the general HF population, mild to moderately severe CKD does not confer the same increased risks of hospitalisation or mortality found in selected or hospitalised HF patients. The risk associated with CKD significantly increases at more severe renal dysfunction or in the presence of other comorbidities. However, worsening renal function during the course of HF is common and identifies HF patients at high risk of imminent hospitalisation and death. Renal dysfunction is an easily accessible, routinely collected and important prognostic tool for HF patients in the general population and with other comorbidities needs to be added to HF optimal management approaches.

## Funding source

This work was supported by (i) a National Institute for Health Research (NIHR, UK) Doctoral Fellowship [grant number NIHR-DRF-2012-05-288]. (ii) Leicester-Wellcome Trust ISSF Fellowship [Reference 204801/Z/16/Z].

## Disclaimers

NIHR: The study sponsors had no role in study design; in the collection, analysis, and interpretation of data; in the writing of the report; and in the decision to submit the paper for publication. The views and opinions expressed therein are those of the authors and do not necessarily reflect those of the NIHR (UK).

CPRD: This study is based in part on data from the Clinical Practice Research Datalink obtained under licence from the UK Medicines and Healthcare products Regulatory Agency. The data is provided by patients and collected by the NHS as part of their care and support. The interpretation and conclusions contained in this study are those of the author/s alone.

HES: Copyright © 2010, re-used with the permission of The Health and Social Care Information Centre. All rights reserved.

## Conflicts of interest

The authors report no relationships that could be construed as a conflict of interest.

## Appendix A Supplementary data

Supplementary tables Image 1
