# Supplementary material for: Chronic kidney disease, worsening renal function and outcomes in a heart failure community setting: A UK national study
Source: Int J Cardiol. 2018 Sep 15;267:120–7. doi: 10.1016/j.ijcard.2018.04.090 (PMC6024224; doi:10.1016/j.ijcard.2018.04.090)
Supplement: Supplementary file 1 — Supplementary tables [file mmc1.docx]

**Table of Contents**

[Table 1 Heart failure selection code set 2](#_Toc505768639)

[Table 2: Dummy code set for chronic kidney disease (CKD) and diabetes 3](#_Toc505768640)

[Table 3: Causal interaction between CKD and other comorbidities in HF 4](#_Toc505768641)

[Table 4: HF population characteristics by renal function; mortality cohort 5](#_Toc505768642)

[Table 5: HF population characteristics by renal function; hospitalisation cohort 6](#_Toc505768643)

[Table 6: HF population characteristics in those with high eGFR (≥90) by mortality outcome 7](#_Toc505768644)

[Table 7: HF population characteristics by worsening renal function (WRF) or any increase in renal function 8](#_Toc505768645)

[Table 8: Renal change effect estimates adjusted for the start and end eGFR 9](#_Toc505768646)

| **Table 1 Heart failure selection code set** | | |
| --- | --- | --- |
| Medcode | Read code | Read term |
| 398 | G580.00 | Congestive heart failure |
| 884 | G581.00 | Left ventricular failure |
| 2062 | G58..00 | Heart failure |
| 2906 | G580.11 | Congestive cardiac failure |
| 4024 | G58z.00 | Heart failure NOS |
| 1223 | G58..11 | Cardiac failure |
| 5942 | G581.13 | Impaired left ventricular function |
| 5255 | G581000 | Acute left ventricular failure |
| 32671 | G580100 | Chronic congestive heart failure |
| 10079 | G580.12 | Right heart failure |
| 9524 | G580.14 | Biventricular failure |
| 17278 | G58z.12 | Cardiac failure NOS |
| 23707 | G580000 | Acute congestive heart failure |
| 10154 | G580.13 | Right ventricular failure |
| 27964 | G582.00 | Acute heart failure |
| 27884 | G580200 | Decompensated cardiac failure |
| 23481 | G581.11 | Asthma – cardiac |
| 43618 | G581.12 | Pulmonary oedema – acute |
| 11424 | G580300 | Compensated cardiac failure |
| 22262 | G58z.11 | Weak heart |
| 12590 | G583.00 | Heart failure with normal ejection fraction |
| 101138 | G580400 | Congestive heart failure due to valvular disease |
| 94870 | G584.00 | Right ventricular failure |
| 104275 | G583.11 | HFNEF - heart failure with normal ejection fraction |
| 101137 | G1yz100 | Rheumatic left ventricular failure |
| 9913 | 101..00 | Heart Failure confirmed |
| 21837 | G232.00 | Hypertensive heart&renal dis wth (congestive) heart failure |

Table 2: Dummy code set for chronic kidney disease (CKD) and diabetes

| Diabetes and CKD were categorised into four mutually exclusive groups of disease combinations |
| --- |
| Diabetes^+^ CKD^-^  Diabetes^-^ CKD^+^  Diabetes^+^ CKD^+^ and  Diabetes^-^ CKD^-^ (reference group) |

The expected risk associated with DM^+^CKD^+^ was calculated by using the equation:

RR Diabetes^+^CKD^+^ (expected) = RR Diabetes^+^ CKD^-^ + RR Diabetes^-^ CKD^+^ – 1

where RR is the Risk Ratio.

| Table 3: Causal interaction between CKD and other comorbidities in HF | | | | | | | |
| --- | --- | --- | --- | --- | --- | --- | --- |
| **Mortality** | | | | **Hospitalisation** | | | |
| **CKD & DIABETES** | | | | | | | |
| Exposure | RR | 95% CI | | Exposure | RR | | 95% CI |
| CKD | 1.16 | 1.11-1.22 | | CKD | 1.12 | | 1.06-1.19 |
| Diabetes | 1.20 | 1.11-1.30 | | Diabetes | 1.20 | | 1.10-1.30 |
| CKD&Diabetes | 1.53 | 1.44-1.62 | | CKD&Diabetes | 1.49 | | 1.39-1.59 |
| RERI 0.16 (0.06 to 0.27) | | | | RERI 0.17 (0.04 to 0.29) | | | |
| S 1.45 (1.09 to 1.93) | | | | S 1.52 (1.05 to 2.2) | | | |
| **CKD & IHD** | | | | | | | |
| Exposure | RR | 95% CI | | Exposure | RR | | 95% CI |
| CKD | 1.18 | 1.11-1.25 | | CKD | 1.13 | | 1.06-1.20 |
| IHD | 1.11 | 1.04-1.18 | | IHD | 1.25 | | 1.16-1.34 |
| CKD&IHD | 1.35 | 1.28-1.43 | | CKD&IHD | 1.47 | | 1.38-1.57 |
| RERI 0.07 (-0.02 to 0.15) | | | | RERI 0.1 (-0.01 to 0.21) | | | |
| S 1.24 (0.91 to 1.68) | | | | S 1.27 (0.96 to 1.68) | | | |
| **CKD & HYPERTENSION** | | | | | | | |
| Exposure | RR | 95% CI | | Exposure | RR | | 95% CI |
| CKD | 1.20 | -0.07-0.11 | | CKD | 1.19 | | 1.10-1.28 |
| Hypertension | 1.16 | 1.08-1.24 | | Hypertension | 1.22 | | 1.14-1.31 |
| CKD&hypertension | 1.38 | 1.30-1.46 | | CKD&Hypertension | 1.38 | | 1.29-1.48 |
| RERI 0.02 (-0.07 to 0.11) | | | | RERI -0.03 (-0.14 to 0.08) | | | |
| S 1.08 (0.85 to 1.36) | | | | S 0.93 (0.72 to 1.20) | | | |
| **CKD & IHD & Diabetes** | | | | | | | |
| Exposure | RR | 95% CI | Exposure | | RR | 95% CI | |
| CKD&Diabetes | 1.32 | 1.23-1.41 | CKD&Diabetes | | 1.30 | 1.19-1.42 | |
| IHD | 1.12 | 1.08-1.16 | IHD | | 1.28 | 1.22-1.34 | |
| CKD&Diabetes&IHD | 1.56 | 1.47-1.66 | CKD&Diabetes&IHD | | 1.78 | 1.63-1.94 | |
| RERI 0.13 (0.01 to 0.25) | | | RERI 0.20 (0.02 to 0.38) | | | | |
| S 1.29 (1.001 to 1.67) | | | S 1.35 (1.03 to 1.78) | | | | |
| CKD, chronic kidney disease defined as eGFR <60 ml/min/m2; IHD, ischaemic heart disease; RERI; relative excess risk due to interaction; S, synergy index.  Red text = significant interaction | | | | | | | |

| Table 4: HF population characteristics by renal function; mortality cohort | | | | | | |  | |  |  |
| --- | --- | --- | --- | --- | --- | --- | --- | --- | --- | --- |
| **Characteristics** |  | |  |  | | |  |  | | |
|  | **≥90 (n=4,091)** | **60-89** (**n=33,772)** | **30-59 (n=54,904)** | | **15-29 (n=11,309)** | **<15(1,996)** |  |  |  |  |
| Age, years | 61[55-66] | 75[67-81] | 81[75-86] | | 84[78-88] | 81[75-87] |  |  |  |  |
| Women | 1,062(26.0) | 12,947(38.4) | 27,331(49.8) | | 6,106(54.0) | 958(48) |  |  |  |  |
| IMD quintile 1 (least) | 341(14.1) | 4,112(20.1) | 7,116(20.9) | | 1,345(19.6) | 221(18.0) |  |  |  |  |
| 5 (most) | 584(24.2) | 3,246(15.9) | 4,885(14.3) | | 998(14.5) | 208(16.9) |  |  |  |  |
| BMI(Kg/m2) | 29[24.7-34] | 27.4[24.0-31.5] | 26.8[23.5-30.7] | | 26.6[23.3-30.5] | 26.4[23.0-30.8] |  |  |  |  |
| Cholesterol(mmol/L) | 4.5±1.2 | 4.5±1.1 | 4.5±1.2 | | 4.4±1.2 | 4.3±13 |  |  |  |  |
| Hb(g/dL) | 13.9±1.8 | 13.4±1.7 | 12.8±1.8 | | 11.8±1.7 | 11.1±1.8 |  |  |  |  |
| Systolic BP(mmHg) | 128.8±19.2 | 130.9±193 | 130.8±20.3 | | 129.6±22.2 | 132.1±24.4 |  |  |  |  |
| Beta blocker | 2,575(62.9) | 19,790(58.7) | 31,645(57.6) | | 6,691(59.2) | 1,181(59.2) |  |  |  |  |
| ACEi or ARB | 3,300(80.7) | 25,768(76.4) | 41,069(74.8) | | 7,204(63.7) | 953(47.8) |  |  |  |  |
| Diuretics | 2,528(61.8) | 23,812(70.6) | 45,948(83.7) | | 10,361(91.6) | 1,621(81.2) |  |  |  |  |
| Aspirin | 2,791(68.2) | 25,238(74.8) | 42,466(77.4) | | 9,071(80.2) | 1,576(79.0) |  |  |  |  |
| Hypertension | 1,883(46.0) | 18,431(54.7) | 34,149(62.2) | | 7,933(70.2) | 1,533(76.8) |  |  |  |  |
| IHD | 1,529(37.4) | 13,884(41.2) | 23,785(43.3) | | 5,044(44.6) | 880(44.1) |  |  |  |  |
| Atrial fibrillation | 1,032(25.2) | 12,307(36.5) | 21,846(39.8) | | 4,313(38.1) | 602(30.2) |  |  |  |  |
| Previous MI | 1,171(28.6) | 9,349(27.7) | 15,231(27.7) | | 3,560(31.5) | 587(29.4) |  |  |  |  |
| COPD | 732(17.9) | 5,367(15.9) | 7,153(13.0) | | 1,387(12.3) | 205(10.3) |  |  |  |  |
| DM | 1,287(31.5) | 8,310(24.6) | 6,714(25.1) | | 14,753(26.9) | 3,711(32.8) |  |  |  |  |
| Smoking yes | 1,082(26.5) | 4,282(12.7) | 4,339(7.9) | | 776(6.9) | 183(9.2) |  |  |  |  |
| Alcohol yes | 3,002(73.4) | 24,328(72.1) | 37,292(67.9) | | 7,378(65.2) | 1,286(64.4) |  |  |  |  |
| Data are number patients(%) or mean±standard deviation or median[IQR]. IMD, index multiple deprivation(1=least deprived, 5=most deprived); BMI, body mass index; Hb, haemoglobin; BP, blood pressure; ACEi, angiotensin-converting enzyme inhibitor; ARB, angiotensin receptor blocker; IHD, ischaemic heart disease; MI, myocardial infarction; COPD, chronic obstructive pulmonary disease; eGFR, estimated glomerular filtration rate.  * National Kidney Foundation Kidney Disease Outcomes Quality Initiative (KDOQI) guidelines. | | | | | | |  |  |  |  |

| Table 5: HF population characteristics by renal function; hospitalisation cohort | | | | | |
| --- | --- | --- | --- | --- | --- |
| **Characteristics** | **≥90 (n=2,859)** | **60-89** (**n=25,588)** | **30-59 (n=38,023)** | **15-29 (n=5,367)** | **<15(562)** |
| Age, years | 61[55-66] | 74[66-81] | 81[75-86] | 84[78-88] | 81[75-87] |
| Women | 785(27.5) | 10,189(39.8) | 20,082(52.8) | 3,212(59.9) | 305(54.3) |
| IMD quintile 1 (least) | 432(15.1) | 5,225(20.4) | 8,067(21.2) | 1,007(18.8) | 127(22.6) |
| 5 (most) | 587(20.5) | 3,787(14.8) | 5,072(13.3) | 728(13.6) | 85(15.1) |
| BMI(Kg/m2) | 29.0[25.1-34.5] | 27.7[24.5-31.8] | 26.9[23.8-30.8] | 26.8[23.6-30.6] | 26.2[23.7-30.7] |
| Cholesterol(mmol/L) | 4.6±1.2 | 4.6±1.1 | 4.7±1.3 | 4.7±1.3 | 4.6±1.2 |
| Hb(g/dL) | 14.2±1.6 | 13.7±1.6 | 13.1±1.7 | 12.0±1.7 | 11.0±1.8 |
| Systolic BP(mmHg) | 131.0±18.3 | 134.8±19.3 | 135.1±20.4 | 135.3±22.3 | 136.6±22.3 |
| Beta blocker | 1,362(47.6) | 11,078(43.3) | 15,840(41.7) | 2,339(43.6) | 255(45.4) |
| ACEi or ARB | 2,342(81.9) | 19,903(77.8) | 29,357(77.2) | 3,650(68.0) | 318(56.6) |
| Diuretics | 1,690(59.1) | 17,190(67.2) | 30,157(79.3) | 4,735(88.2) | 473(84.2) |
| Aspirin | 1,818(63.6) | 17,246(67.4) | 26,949(70.9) | 4,072(75.9) | 401(71.4) |
| Hypertension | 1,390(48.6) | 13,497(52.8) | 23,479(61.8) | 3,806(70.9) | 410(73.0) |
| IHD | 855(29.9) | 8,869(34.7) | 13,812(36.3) | 2,018(37.6) | 215(38.3) |
| Atrial fibrillation | 728(25.5) | 7,995(31.3) | 13,519(35.6) | 1,730(32.2) | 117(20.8) |
| Previous MI | 716(28.6) | 5,698(22.3) | 8,197(21.6) | 1,213(22.6) | 156(27.8) |
| COPD | 364(12.7) | 3,187(12.5) | 3,802(10.0) | 505(9.4) | 46(8.2) |
| DM | 827(28.9) | 5,602(21.9) | 8,574(22.6) | 178(31.7) | 178(31.7) |
| Smoking yes | 735(25.7) | 3,010(11.8) | 3,069(8.1) | 375(7.0) | 52(9.3) |
| Alcohol yes | 2,243(78.5) | 19,758(77.2) | 27,364(72.0) | 3,557(66.3) | 359(63.9) |
| Heart failure admission prior to CPRD HF index date | |  |  |  |  |
| Yes | 389(13.6) | 2,949(11.5) | 5,492(14.4) | 1,217(22.7) | 161(28.6) |
| Hospital admission during 1-year prior to CPRD HF index date | | |  |  |  |
| 0-3 months before | 193(6.8) | 1,790(7.0) | 2,891(7.6) | 657(12.2) | 124(22.1) |
| 3-6 months before | 274(9.6) | 2,187(8.5) | 3,097(8.1) | 435(8.1) | 58(10.3) |
| 6 to 12 months before | 429(15.0) | 3,059(12.0) | 4,711(12.4) | 678(12.6) | 72(12.8) |
| Data are number patients(%) or mean±standard deviation or median[IQR]. IMD, index multiple deprivation(1=least deprived, 5=most deprived); BMI, body mass index; Hb, haemoglobin; BP, blood pressure; ACEi, angiotensin-converting enzyme inhibitor; ARB, angiotensin receptor blocker; IHD, ischaemic heart disease; MI, myocardial infarction; COPD, chronic obstructive pulmonary disease; eGFR, estimated glomerular filtration rate. * National Kidney Foundation Kidney Disease Outcomes Quality Initiative (KDOQI) guidelines. | | | | | |

| Table 6: HF population characteristics in those with high eGFR (≥90) by mortality outcome | | |
| --- | --- | --- |
|  | | |
| **Characteristics** | **Case (n=494)** | **Control** (**n=3,627)** |
| Age, years | 65 [60 to 71] | 61 [55 to 66] |
| Women | 137(29.5) | 925(25.5) |
| IMD quintile 1 (least) | 39(8.4) | 302(8.3) |
| 5 (most) | 71(15.3) | 513(24.1) |
| BMI(Kg/m2) | 26.4 [22.3 to 30.7] | 29.4 [25.1 to 24.3] |
| Cholesterol(mmol/L) | 4.5±1.3 | 4.5±1.1 |
| Hb(g/dL) | 13.0±2.2 | 14.0±1.7 |
| Systolic BP(mmHg) | 123.5±21.7 | 129.5±18.7 |
| Beta blocker | 184(39.7) | 2,397(65.9) |
| ACEi or ARB | 273(58.8) | 3027(83.5) |
| Diuretics | 329(70.9) | 2199(60.6) |
| Aspirin | 307(66.2) | 2484(68.5) |
| Hypertension | 285(61.4) | 1,923(53.0) |
| IHD | 177(38.1) | 1,352(37.3) |
| Atrial fibrillation | 121(26.1) | 911(25.1) |
| Previous MI | 125(26.9) | 1,046(28.8) |
| COPD | 155(33.4) | 577(15.9) |
| DM | 148(31.9) | 1,139(31.4) |
| Smoking yes | 160(34.5) | 923(25.4) |
| Alcohol yes | 329(70.9) | 2,685(74.0) |
| Data are number patients(%) or mean±standard deviation or median[IQR]. IMD, index multiple deprivation(1=least deprived, 5=most deprived); BMI, body mass index; Hb, haemoglobin; BP, blood pressure; ACEi, angiotensin-converting enzyme inhibitor; ARB, angiotensin receptor blocker; IHD, ischaemic heart disease; MI, myocardial infarction; COPD, chronic obstructive pulmonary disease; eGFR, estimated glomerular filtration rate.  * National Kidney Foundation Kidney Disease Outcomes Quality Initiative (KDOQI) guidelines. | | |

| Table 7: HF population characteristics by worsening renal function (WRF) or any increase in renal function | | | | | | |
| --- | --- | --- | --- | --- | --- | --- |
|  | **Mortality** | | | **Hospitalisation** | | |
| **Characteristics** | WRF (>20% loss)  n=18,709 | Any increase  n=37,484 | No change  n=13,434 | WRF ( >20% loss)  n=11,249 | Any increase  n=23,017 | No change  n=7,448 |
| Age, years | 80[74-86] | 79[71-85] | 78[70-84] | 80[73-85] | 78[71-84] | 78[70-84] |
| Women | 8,854(47.3) | 17,094(45.6) | 5,785(43.1) | 5,407(48.1) | 10,798(46.7) | 3,439(46.2) |
| IMD quintile |  |  |  |  |  |  |
| 1 least | 2,358(20.5) | 4,676(20.2) | 1,650(19.8) | 2,331(20.7) | 4,733(20.5) | 1,567(21.0) |
| 5 most | 1,711(14.9) | 3,590(15.2) | 1,180(14.2) | 1,471(13.1) | 3,325(14.4) | 1,036(13.9) |
| Beta blocker | 11,231(60.0) | 22,906(61.1) | 8,214(61.1) | 5,006(44.5) | 10,656(46.1) | 3,427(46.0) |
| ACEi or ARB | 13,998(74.8) | 27,660(73.8) | 10,427(77.6) | 9,130(81.2) | 18,446(79.8) | 5,897(79.2) |
| Diuretics | 16,680(89.2) | 28,684(76.5) | 10,125(75.4) | 9,458(84.1) | 17,195(74.4) | 2,075(27.9) |
| Aspirin | 14,784(79.0) | 29,601(79.0) | 10,464(77.9) | 8,271(73.5) | 16,849(72.9) | 5,421(72.8) |
| BMI(Kg/m2) | 26.9[23.9-31.4] | 27.1[23.7-31.2] | 27.3[23.9-31.4] | 27.3[24.0-31.4] | 27.4[24.2-31.5] | 27.4[24.4-31.4] |
| Cholesterol(mmol/L) | 4.4±1.2 | 4.4±1.1 | 4.4±1.1 | 4.6±1.2 | 4.6±1.2 | 4.6±1.1 |
| Hb(g/dL) | 12.3±1.9 | 12.9±1.8 | 13.2±1.8 | 12.8±1.8 | 13.2±1.7 | 13.4±1.7 |
| Systolic BP(mmHg) | 127.7±21.5 | 130.7±19.6 | 131.4±1.8 | 132.5±20.8 | 1347±20.0 | 135.3±19.0 |
| Most recent eGFR | 37.7±15.8 | 58.9±19.5 | 59.3±19.8 | 44.5±16.4 | 58.7±18.8 | 59.6±18.9 |
| 1: ≥90 | 3(0.0) | 2,142(5.7) | 914(6.8) | 34(0.3) | 1,242(5.4) | 508(6.8) |
| 2: 60-89 | 17,38(9.3) | 15,388(41.1) | 5,328(39.7) | 2,002(17.8) | 9,356(40.5) | 2,954(39.7) |
| 3: 30-59 | 10,701(57.2) | 1,7482(46.6) | 6,356(47.3) | 6,917(61.5) | 1,1113(48.1) | 3,628(48.7) |
| 4: 15-29 | 4,964(26.5) | 2,211(5.9) | 785(5.8) | 2,006(17.8) | 1,310(5.7) | 341(4.6) |
| 5: <15 | 1,303(7.0) | 261(0.7) | 51(0.4) | 290(2.6) | 86(0.4) | 17(0.2) |
| Previous eGFR | 53.1±19.4 | 52.0±18.5 | 60.1±20.3 | 55.6±18.7 | 52.8±17.9 | 61.1±19.5 |
| 1: ≥90 | 550(2.9) | 930(2.5) | 1,287(9.6) | 406(3.6) | 528(2.3) | 663(8.9) |
| 2: 60-89 | 6,348(33.9) | 11,54730.8() | 55,32(41.2) | 4,189(37.2) | 7447(32.2) | 3085(41.4) |
| 3: 30-59 | 9674(51.7) | 20,844(55.6) | 59,16(44.0) | 5792(51.5) | 12809(55.4) | 3399(45.6) |
| 4: 15-29 | 1854(9.9) | 3,733(10.0) | 6,55(4.9) | 808(7.2) | 2184(9.5) | 288(3.9) |
| 5: <15 | 283(1.5) | 4,30(1.1) | 44(0.3) | 54(0.5) | 139(0.6) | 13(0.2) |
| Hypertension | 12,311(65.8) | 23,402(62.4) | 8,077(60.1) | 7,132(63.4) | 14,363(62.2) | 4,598(61.7) |
| IHD | 8,388(44.8) | 16,745(44.7) \| | 7,493(55.8) | 4,114(36.6) | 8,826(38.2) | 2,870(38.5) |
| Previous MI | 5,465(29.2) | 11,006(29.4) | 39,39(29.3) | 2,398(21.3) | 5,644(24.4) | 1,761(23.6) |
| AF | 7,554(40.4) | 14,663(39.1) | 4,931(36.7) | 3,974(35.3) | 7,849(34.0) | 2,450(32.9) |
| COPD | 2,662(14.23) | 5,478(14.6) | 1,915(14.3) | 1,243(11.1) | 2,591(11.2) | 811(10.9) |
| Diabetes | 6,513(34.8) | 10,793(28.8) | 3,3665(27.3) | 3,189(28.4) | 6,141(25.6) | 1,881(25.3) |
| Current smoker | 1,588(8.5) | 3,527(9.4) | 1,346(10.0) | 1,045(9.3) | 2,094(9.1) | 675(9.1) |
| Current alcohol | 12,616(67.4) | 25,864(69.0) | 9,461(70.4) | 5,059(73.3) | 15,760(73.2) | 7,491(72.3) |
| IMD, index multiple deprivation (1=least deprived, 5=most deprived); BMI, body mass index; Hb, haemoglobin; BP, blood pressure; ACEi, angiotensin-converting enzyme inhibitor; ARB, angiotensin receptor blocker; IHD, ischaemic heart disease; MI, myocardial infarction; COPD, chronic obstructive pulmonary disease; eGFR, estimated glomerular filtration rate (ml/min/m2)); WRF, worsening renal function.  For all-cause mortality, WRF was calculated over a year before the match date using the most recent value (up to a maximum of 1 year) and a second value between 3 months and 2 years earlier. For all-cause hospital admission, WRF was calculated over 6-months before the match date using the most recent value (up to a maximum of 6-months) and a second value between 3-months and 1 year earlier. | | | | | | |

| **Table 8: Renal change effect estimates adjusted for the start and end eGFR** | | | | | | |  |
| --- | --- | --- | --- | --- | --- | --- | --- |
|  | **All-cause mortality** | | | **All-cause first hospital admission** | | | |
|  | **Adjusted** | **Adjusted for start eGFR** | **Adjusted for end eGFR** | **Adjusted^3^** | **Adjusted for start eGFR** | **Adjusted for end eGFR** | |
|  | **OR (95% CI)** | **OR (95% CI)** | **OR (95% CI)** | **OR (95% CI)** | **OR (95% CI)** | **OR (95% CI)** | |
| eGFR change |  |  |  |  |  |  | |
| 0-5% decrease (ref) | 1.0 | 1.0 | 1.0 | 1.0 | 1 | 1 | |
| >20% decrease (WRF) | 1.92(1.79-2.06) | 1.88 (1.76-2.02) | 1.74 (1.62-1.87) | 1.50(1.37-1.64) | 1.49(1.36-1.62) | 1.43(1.31-1.57) | |
| 6-20% decrease | 1.12(1.04-1.20) | 1.10 (1.03-1.18) | 1.08 (1.00-1.15) | 1.11(1.02-1.21) | 1.10(1.01-1.20) | 1.09(0.99-1.22) | |
| Any % increase | 1.22(1.15-1.31) | 1.18 (1.10-1.26) | 1.24 (1.16-1.32) | 1.13(1.04-1.22) | 1.11(1.02-1.20) | 1.12(1.04-1.22) | |
| eGFR, estimated glomerular filtration rate (ml/min/m^2^); ref, reference group. All associations were fully adjusted by all remaining covariates and the start or end eGFR.. For all-cause mortality outcome, change was calculated over a year before the match date using the most recent value up to a maximum of 1 year and an earlier value between 6 months and 2 years prior. For all-cause first hospital admission outcome, change was calculated over 6-months before the match date using the most recent value up to a maximum of 6-months and an earlier value between 3-months and 1 year prior. | | | | | | | |
